# Living Arrangement: a Contributor to Vascular Disease in Asymptomatic African American Women

**DOI:** 10.20429/jgpha.2019.070220

**Published:** 2019

**Authors:** Meldra Hall, Elizabeth Ofili, Rigobert Lapu-Bula, Ernest Alema-Mensah, Stephanie Miles-Richardson

**Affiliations:** 1 Master of Public Health Program, Morehouse School of Medicine, Atlanta, GA; 2 Clinical Research Center, Morehouse School of Medicine, Atlanta, GA

**Keywords:** Living arrangement, social support, vascular disease, arterial elasticity, vascular function

## Abstract

**Background:**

Diminished social support lias shown to lead to worse cardiovascular outcomes and since cardiovascular disease (CVD) is the leading cause of death in the United States (U.S.), it is critical to non-invasively study its precursor- vascular disease (VD). Assessing the impact social support lias on vascular outcomes can unveil potential CVD susceptibilities in at-risk populations. African American women exhibit the greatest burden of CVD morbidity and mortality; therefore, the purpose of tins study is to examine the association between living arrangement/social support and impaired vascular function in asymptomatic African American women.

**Methods:**

Vascular function was assessed by a non-invasive screening tool, HDI/PulseWave CR-2000, during screenings at community outreach events on participants clinically free of CVD. Vascular disease was defined as abnormal/impaired vascular function. Living arrangement, a binary variable (living with someone/living alone), was determined by survey responses (N=67) and represented social support. Multivariable analyses were used to estimate adjusted odds ratios (AORs) and 95% confidence intervals (95% CIs) to determine the association between living arrangement and vascular disease after controlling for confounders. Analyses were conducted using SAS 9.2.

**Results:**

Of those who lived alone, 82% had vascular disease (p=0.03). After adjusting for family CVD, and other CVD risk factors, those who lived with a spouse/partner or relative were 78% (p=0.04) less likely to develop vascular disease (AOR=0.22; 95% 0=0.05, 0.98).

**Conclusions:**

Our study provides preliminary evidence to suggest that among African American women, clinically free of CVD, living arrangement is associated with vascular disease. While living alone may place individuals at an increased risk of CVD because of the association, living with a spouse/partner or relative may act as a protective factor against vascular disease and reduce the risk of CVD. Public health practitioners may use individuals’ living arrangement as preventive measure for CVD risk.

## INTRODUCTION

Since cardiovascular disease (CVD) is a leading cause of death globally, it remains a major public health concern worldwide. In the US, about 25% of deaths are due to CVD annually (Centers for Disease Control and Prevention [CDC], 2015) and 1.9% of people live with disability from CVD (Murray, 2013). In addition to the annual death toll, more than 2,150 Americans die each day in the US from CVD (Mozaffarian, 2015).

Furthermore, the Southeast (SE) region is observed to have the highest rates of CVD deaths compared to other regions in the United States (Mozaffarian, 2015). The following include states in the SE region of the U.S. along with the number of heart disease deaths per 100,000: Alabama (222.5), Arkansas (223.7), Florida (146.2), Georgia (179.0), Kentucky (203.0), Louisiana (213.1), Mississippi (233.1), North Carolina (155.8), South Carolina (173.8), Tennessee (198.8) and Virginia (150.7) (CDC, 2018). Georgia’s death rate at 179 per 100,000 deaths is higher than the national rate (165.5 deaths per 100,000), along with 8 of the other southeastern states: Alabama, Arkansas, Florida, Kentucky, Louisiana, Mississippi, South Carolina, and Tennessee (CDC, 2018). Of the SE states, 9 of the 11 (82%) have significantly worse heart disease outcomes compared to the national rate.

CVD accounts for 25% of deaths in women (CDC, 2015). Despite the fact that CVD is the leading cause of death for women in the United States, 46% of women do not recognize their risk (CDC, 2015).

Moreover, a disproportionate burden of CVD rests with African Americans. The risk of CVD deaths in African Americans is significantly greater than the risk in Caucasians, Asians, and American Indians (CDC, 2015). While African Americans make up 13.2 % of the US population, CDC’s estimates show that 23.8% of all reported CVD deaths are African American (CDC, 2015). African Americans are 33% more likely to be diagnosed with CVD than their non-minority counterparts (CDC, 2015). Although CVD trends in mortality have shown a reduction for many racial groups, deaths rates have not decreased for the African American population. Non-Hispanic blacks are 1.6 times more likely to die of CVD than non-Hispanic whites (Lang & Bird, 2015).

Specifically, heart disease significantly impacts African American women. Black women have a 41% greater risk of CVD mortality than white women (Coulter, 2011). From 2000-2006, there were 566 per 100,000 black women whose deaths were attributable to CVD while 416 per 100,000 white women and 332 per 100,000 Hispanic women died from CVD (Coulter, 2011). Unfortunately, racial/ethnic and gender disparities in CVD mortality continue (Coulter, 2011) and research shows that African American women continue to bear the greatest burden of disease (AHA, 2014).

A better understanding of risk factors for CVD as assessed by vascular function is paramount for early detection and prevention of CVD.

Vascular function is referred to as the arterial compliance (i.e., arterial elasticity or distensibility or capacitance or stiffness) of the arterial wall as the blood flows through the blood vessel, which distends and contracts to enable blood to move directionally (John Hopkins, 2015). Noninvasive techniques can be used to examine arterial compliance, peripheral pressure, and vascular tonometry (Duprez, 2004). The relationship between waveform detecting sensors and arterial stiffness is vital to understanding arterial health (Duprez, 2010). Examining vascular function among an at-risk population has public health implications because early detection of vascular disease (VD) in asymptomatic individuals with CVD can serve as primary prevention and is essential to identifying future cardiac events. Increased arterial stiffness or decreased arterial elasticity (distensibility) is an independent predictor of CVD risk and mortality (Duprez, 2004).

This screening device [HDI/CR-2000 CVProfiler] is used particularly to detect changes in vascular dimensions (DeLoach and Townsend, 2008) [via C1 and C2]. It is specifically advantageous because it noninvasively quantifies peripheral arterial stiffness in both large (C1) and small (C2) arteries using radial pulse tonometry (Duprez, 2004 and Duprez, 2010)].

C1 and C2 have shown to be inversely associated with index of arterial stiffness (i.e., augmentation index) and were strongly associated with the development of arterial hypertension (Duprez, 2010) and incident CVD (Duprez, 2011). They may therefore serve as early independent indicators predicting CVD risk and mortality (Cohn, Duprez 2004/2011, and Grandits, 2005, Wilson 2004) because of their association with body composition (Forbang, 2015).

Another study also examined vascular function and the authors noted that physiological conditions affect arterial elasticity (Tripolino, 2016). Although Tripolino et al. primarily focused on blood viscosity, they noted that elasticity of arterial wall serves as a major contributor to vascular events (Tripolino, 2016).

In the Multi-ethnic Study of Atherosclerosis (MESA) study, cardiovascular risk factors along C1 and C2 were compared in four race/ethnic groups and African Americans tended to have a more impairment in artery elasticity than other ethnic groups including Chinese, Hispanics and Caucasians (Duprez, 2009 and Hall, 2012). In this MESA study, an association of small artery elasticity with incident CVD was found, suggesting that the analysis of microvasculature such as small arteries could be beneficial for the prediction of early clinical cardiovascular events (Duprez, 2011).

The influence of living arrangement on vascular disease remains not fully understood. Limited studies assessing vascular function in at risk but asymptomatic African American women have shown conflicting results and none has specifically examined the impact of living arrangement on vascular disease in a population of African American women from the southeast region of the US who are at risk but asymptomatic for CVD. A better understanding of risk factors for VD such as living arrangement could provide an opportunity for early detection and prevention of VD and, ultimately CVD.

Trends in the literature show that for minority populations, older minorities are more likely to live alone (Himes, 1996). Although blacks more often live with extended family households, (Goldscheider and Bures, 2003), Himes et al found that nine out of ten elderly minority women live alone (Himes, 1996).

Living alone serves as an indicator for social isolation and diminished social support (Case, 1992). A study conducted by Kitamura et al. evaluated the living arrangements of patients who had been discharged from the hospital after having an acute myocardial infarction (Kitamura, 2013). They compared those who lived alone with those who lived with a spouse or relative. The study found that living arrangement, particularly, living alone, is independently associated with an increased risk of cardiovascular disease that living alone led to adverse cardiovascular events (Kitamura, 2013). Because of the reduced social support, living alone leads to increased rates of mortality after a heart attack (Kitamura, 2013). Living alone is an independent risk factor for CVD after an acute myocardial infarction (Schmaltz, 2007). Moreover, Brutto et al. revealed through their findings that social isolation was detrimental to an individual and it lead to worse cardiovascular health when compared to their counterparts who lived with someone (Brutto, 2013). Researchers suggested that individuals living alone should be given more attention (i.e. support) so as to allow for secondary prevention (Kitamura, 2013). Understanding living arrangements provides an opportunity to evaluate measurable psychosocial variables that may lead to adverse health outcomes.

This study aims to determine the impact living arrangement has on vascular disease. This study fills the gap in literature by addressing the effect of living arrangement on vascular disease specifically in African American women from the Southeast region.

Although studies have examined vascular disease in African American women, the participants included in other studies came to the laboratory, clinic, or testing site. This study is unique in that the screenings occur in the community by intentionally bringing the vascular screening instrument to a community of individuals who are free of symptomatic cardiovascular disease.

Since there is a gap in the literature that focuses on the impact of living arrange on vascular disease, the purpose of this study is to examine the association between living arrangement and vascular disease, as assessed by C1 and C2 in African American women residing in the Southeast region of the United States.

Therefore, the research question for this study is ‘Does living arrangement have an association with vascular disease among African American women residing in the southeast region of the US who are asymptomatic for cardiovascular disease?’ We hypothesize that there will be an association between living arrangement and vascular disease as assessed by C1 and C2.

## METHODS

### Procedures

The inclusion criteria for this study included being female, being of African descent/African American, residing in the Southeast region of the United States, and having no known CVD. Individuals were excluded if they were male, had been clinically diagnosed with cardiovascular disease, under the age of 18, and lived outside of the Southeast region of the United States.

This study is a cross-sectional study designed to assess the relationship between living arrangement and vascular disease. Recruitment for this study was conducted at the Links Southeast Regional Conference in Birmingham, Alabama; Impact Church, East Point Georgia; and H.J.C. Bowden Senior Multipurpose Facility, in Atlanta Georgia. The sample size is 67. This study design was approved by the Institutional Review Board at Morehouse School of Medicine.

### Recruitment

Recruitment for this study was conducted by convenience sampling from various community sites. Convenience sampling is one of the main types of non-probability sampling methods and is utilized when the sample is made up of individuals who are easy to reach. Community sites were selected due to partnerships that were established with the Morehous School of Medicine Clinical Research Center.

The methodology of this study is unique in that participants did not come to a research lab; rather, we intentionally sought out individuals who were in the community. Investigators included cardiovascular researchers, a research nurse, a technician, a cardiac sonographer and a public health student from the Clinical Research Center at Morehouse School of Medicine. This study was approved by the Institutional Review board at Morehouse School of Medicine.

Because we were going into the community, we used the mobile clinical research unit owned by Morehouse School of Medicine as the site where we conducted clinical testing. The Mobile Unit currently holds two areas where clinical testing can be conducted.

### Study participants/study sites

Study participants were all female and identified themselves as African American. These community participants were clinically free of incident, diagnosed or symptomatic CVD. Signed informed consent was obtained by all participants at all sites after the nature of the study had been explained.

#### Study site 1:

The mobile unit was taken to the first community study site at the Links Conference in Birmingham, Alabama on May 15, 2015 (n=38). The Links Incorporated, is an organization made up of women of African descent. These women are educated, have professional accomplishments and professional occupations. Of the women screened, 90% had obtained a doctoral level degree. This organization works to ensure the identities, culture, and economic survival of all people of African origin through cultural, educational, and civic programs through philanthropic events. Data were collected from participants who were members of The Links, Incorporated organization at a Links Conference community event.

The age range for women in this sample was from 38years to 79 years. Seven states in the southeast region of the United States (Alabama, Georgia, Mississippi, South Carolina, North Carolina, Louisiana, and Florida) were represented. These women were all community members.

#### Study site 2.

The Impact Church, in Atlanta, GA, was the second community site where data were collected on September 27, 2015 (n=20). The individuals who participated at this site were church members. Impact church is a multicultural gathering of people. Events are held by church members throughout the year. This church is technologically advanced and its members use their portable devices with consistency. They had an age range from 45-65 years and were from the community.

#### Study site 3.

The H.J.C. Bowden Senior Multipurpose Facility, in Atlanta, GA, was the third community site where data were collected on December 8, 2015 (n=9). It is Fulton County’s largest senior activity center in District 6. It is a place where individuals over the age of 54 can come for physical activity and enjoy amenities to enhance their mind, body, and spirit.

These individuals pay for breakfast and lunch at this facility; they have access to a fitness room, multipurpose room, therapeutic pool for arthritic joints, and services from a physician who specializes in geriatrics. These community members are elderly, but are physically active. Individuals can afford additional amenities, and therefore from a higher socioeconomic status.

### Data collection/measurement tool

Two instruments were used to collect data: a survey and vascular screening tool. The survey included paper and electronic versions. The survey instrument for cardiovascular risk assessment has been previously validated (Cohn, J., Duprez D., and Grandits G., (2005). Surveys completed with a paper version were imported into REDCap. Electronic versions were completed by participants in a REDCap survey. Data on living arrangements as well as family history and other risk factors were collected in the survey. The screening tool that was utilized in this study is the HDI/ Pulsewave-CR 2000 (Hypertension Diagnostics Inc. device). The vascular screening tool was used to determine if vascular disease was present.

The survey was the instrument used to collect and measure the living arrangement variable. Living arrangement was defined as a binary variable categorized as living alone or living with a spouse/partner or relative. If participant indicated that she lived with a spouse, partner, or relative (i.e. ‘Living with spouse/partner or relative’), they were classified as living with someone. If the participant indicated that she did not live with a spouse, partner, relative or other, then she was placed in the category: ‘living alone’. Socioeconomic status was defined by level of education which was measured by survey responses.

In addition to living arrangement, the survey measured both family history of CVD and personal risk factors. Family history was measured by having a mother, father, paternal grandfather, paternal grandmother, maternal grandfather, maternal grandmother or siblings who had been diagnosed with cardiovascular disease. Cardiovascular disease was defined as having at least one of the following: high blood pressure, heart attack, heart failure, stroke, or angina. This instrument incorporated questions, such as, “Do you know your father’s health history?” If the responder answered ‘yes’, follow-up questions asked about “high blood pressure”, “heart attack”, “heart failure”, “stroke”, and “angina”. Personal risk factors were measured by having hypertension, diabetes, high lipid cholesterol, or using tobacco. A response of ‘yes’ or ‘no’ was required. If the response was ‘yes’ the individual was identified as having that heart condition and if the response was ‘no’, the individual was identified as not having that heart condition. The second instrument, the HDI/PulseWave CR-2000, (Hypertension Diagnostics, Inc., Eagan, Minnesota) was used to measure vascular disease by detecting C1 and C2 biomarkers of arterial elasticity. This radial tonometry device is unique in that it quantifies the large (C1) and the small (C2) artery elasticity indices for assessment of arterial compliance. The instrument noninvasively detected radial arterial pulse waves while participant’s hand rested in the supine position (Duprez, 2004). Additionally, this instrument can detect vascular disease in individuals who are not clinically diagnosed with CVD- they have no signs or symptoms of CVD; however, the tool identifies disease in the artery.

The computer screen records waveforms are detected by screening probe. The waveforms were applied to a modified Windkessel model for calculation of C1 and C2, small and large artery indices, respectively (Duprez, 2004).

### Statistical analysis

Primary data analysis was run using SAS version 9.2 to obtain results. A univariate analysis was conducted to characterize sample population and obtain descriptive analysis. The descriptive statistics were run for living arrangement, Family CVD, personal risk factors, level or education, and arterial elasticity (C1 and C2).

The bivariate analysis used Fisher’s exact test to examine the association between living arrangement and vascular disease as assessed by C1 and C2. Also, bivariate analysis was used to examine the associations between other confounders and vascular disease.

A multivariable regression analysis was conducted to examine the inherent association between living arrangement and vascular disease after controlling for any confounders and risk factors noted in the literature.

We estimated the odds ratio at a 95% confidence interval for living arrangement, risk factors, family CVD, and grandparent CVD. Statistical significance was determined at p<= 0.05. The power of the sample size was estimated. The statistical software we used was SAS 9.2.

## RESULTS

### Univariate analysis

Results from univariate analysis showed descriptive statistics (Table 1). A total of 67 participants made up the study sample. Of those who were screened (n=46), 18 had normal/borderline screening and 28 had abnormal screening. Participants (n=67) completed the survey but were unable to be screened due to the closing of the site/facility. For living arrangement, 20 participants lived alone while 47 lived with a spouse/partner or with a relative.

### Bivariate Analysis

Bivariate analysis included Family CVD, Mom-Dad CVD, living arrangement, and tobacco use crossed with arterial elasticity screening (Table 2). Arterial Elasticity Screening was divided into: ‘Normal/Borderline’ and ‘Abnormal’ vascular screening. Abnormal vascular screening reflects standardized values that correspond with an individual’s gender and age is and indicative of vascular disease.

### Living arrangement crossed with arterial screening C1

Living arrangement was categorized into ‘Living Alone’ and ‘Living with Spouse/Partner or with Relative’. Of the individuals that lived alone, five had normal/borderline screening while 12 had diseased arteries. In other words, of those living alone, 29% screened normal/borderline while 71% had vascular disease. Of the 29 individuals living with a spouse, partner, or relative, 13 had normal/borderline arterial screening while 16 had abnormal screening. That is, of those living with someone, 45% had a normal/borderline screening while 55% had an abnormal screening for arterial elasticity. Statistical significance was determined at p<=0.05.

### Living arrangement crossed with arterial screening C2

Living arrangement was divided into ‘Living alone’ and ‘Living with Spouse/Partner or with Relative’. Of the participants who lived alone, three had normal/borderline vascular screening while 14 had vascular disease. Of those living with a spouse/partner or relative, 15 were normal/borderline arterial wall and 14 had vascular disease. For C2 arterial screening, 82% of those living alone had abnormal vascular screening. Note that the number of those who had normal/borderline screening was slightly greater than those who did not.

### Multivariable Analysis

Multivariable regression analysis was run to adjust for confounders and risk factors noted in the literature, which included family CVD, and personal risk factors, which were defined as hypertension, tobacco use, diabetes, and high lipid cholesterol.

### C1 arterial screening

Living arrangement was crossed with C1 arterial elasticity screening. ‘Living Alone’ was the reference group. Those who were living with a spouse/partner or relative were 65% less likely to have vascular disease. For family history of CVD, ‘No heart condition’ was the reference group. Those who had heart disease in their family were 90% more likely to have vascular disease at p=0.05. For high lipid cholesterol, ‘No high cholesterol’ was the reference group.

### C2 arterial screening

Living arrangement was crossed with C2 arterial elasticity screening. ‘Living Alone’ was the reference group. Those who lived with a spouse, partner, or relative were 78% less likely to have vascular disease at p=0.04. Family history was associated with abnormal vascular screening.

## DISCUSSION

Although the results had borderline p-values, the findings from this study suggest that for C2, which was a measure of vascular disease, there is a significant association with living arrangement among African American women free of incident or symptomatic cardiovascular disease living in the Southeast region of the U.S. In our study, 82% of individuals who lived alone had vascular disease and after adjusting for potential confounders and risk factors, individuals who live with a spouse, partner, or relative were 65% less likely to have vascular disease when C1 is measured and 78% less likely when measuring C2. Living with someone may serve as a protective factor against vascular disease.

Social isolation can lead to adverse health outcomes (Li, 2006) and insufficient social support has proven to have an impact on cardiovascular outcomes (Brutto, 2013). Since living with someone can reduce social isolation and increase the social support, an individual living with someone provides a social structure within the home that nurtures an environment where individuals can be less vulnerable to the development of CVD. Our findings suggest that living arrangement is associated with primary vascular disease.

This study is not without limitations. The study design used in this research was a cross-sectional study design. Cross-sectional studies are a snapshot of the relationship between the exposure variable and the outcome variable- it cannot establish causality. Another limitation is the sample size. This study is in the early stages, and therefore has a small sample size. This study has limited generalizability. It may not be representative of all African American women because it does not account for the various ethnicities represented by the African American race. Education level could be a confounder since 90% of the women in the first screening site had obtained a doctoral degree; this could have skewed the data. The living arrangement variable does not account for those who live with non-relatives (i.e. friend). Lastly, this study does not account for social support structure from outside of the home.

Despite these limitations, this study has strengths and characteristics that make it unique. For instance, this study is inclusive of community members- individuals who did not come to a research facility. The participants in this study are clinically free of cardiovascular disease. Lastly, vascular disease was assessed by well-defined vascular screening instrument. The results from this study can afford opportunities for future studies that target this particular population.

## CONCLUSIONS

Our study provides preliminary evidence to suggest that among African American women who are clinically free of CVD and residing in the Southeast region of the US, living arrangement is associated with vascular disease as assessed by C2.

This is the first study to report a relationship between living arrangement and vascular disease among asymptomatic African American women in the Southeast region of the United States. The association of living arrangement along with family history of cardiovascular disease, parent CVD, and current or past tobacco use with vascular disease has directionality. Living with someone or having supplemental social support may reduce the risk of a cardiovascular event. Because vascular disease is a precursor to cardiovascular disease, early detection of vascular disease is vital for the prevention of CVD. Vascular disease can be assessed by biomarkers, C1 and C2. Abnormal C1, and specifically C2, indicate the presence of disease. C1 independently predicts future cardiovascular disease and C2 is associated with the risk of cardiovascular disease. Not only do these results reveal the factors that are inherently associated with vascular disease, these results show that living with someone is inversely associated with vascular disease.

Early detection of vascular disease affords an opportunity to prevent cardiovascular disease. This screening tool serves as a method of prevention for at risk populations. Because more individuals in the target population fall victim to cardiovascular disease than any other illness in the United States, preventing incident CVD by detecting it early will prevent CVD mortality within this population.

The current guidelines for vascular screenings to detect CVD are blood pressure measurements which include multiple reads (3) for accuracy and electrocardiograms (Wallace, 2015). These screenings, along with treatment and lifestyle changes due to the information that the screening provide have been shown to significantly reduce the occurrence of CVD events.

Living alone has been shown to be associated with vascular disease which is a precursor of cardiovascular disease. Individuals who live in a residence with no one are more likely to have vascular disease and therefore, as the literature shows, are more likely to be at risk for cardiovascular events, such are heart attacks and strokes.

The HDI/CR-2000 is a unique screening tool because of its ability to detect vascular disease by measuring large and small artery elasticity in asymptomatic individuals. Vascular tonometry serves as a vital tool in predicting the presence of vascular disease. The use of a vascular screening tool allows for the early identification of vascular disease, fosters early detection before symptoms appear, allows for easier treatment before the onset of cardiovascular disease, may prevent future cardiac events, and may lead to a lower risk of CVD death. Because of these affirmative outcomes, individuals with identified risk factors should be afforded an opportunity to be screened for vascular disease.

Moreover, it is important to understand the disease among community members. This study makes use individuals clinically free of cardiovascular disease and who have unsuspected vascular disease; nevertheless, this screening tool is able to detect disease within the blood vessel.

The study has implications for population wide prevention strategies in at risk African American women; knowing vascular disease in asymptomatic at-risk women can allow for early detection of cardiovascular disease intervention before symptoms appear. More so, there is a need to address gender and racial issues pertaining to CVD outcomes.

The study is in the early stages and has provided preliminary evidence. Future research should gather more data to increase sample size which will lead to greater statistical significance; should use a survey instrument that more clearly defines social support to identify if relationships still contribute to the social support independent of the individual living in the same house; and should more closely examine the mechanisms by which living alone can impact vascular disease.

Public health practitioners should integrate prevention strategies for those who are at risk for cardiovascular disease and create a program that provides social support to at risk individuals. Someone present in the house provides social support and serves as accountability, and decisions that affect health outcomes may be more intentional. Public health and clinical researchers should conduct more vascular screenings in an at-risk population. Public Health organizations can partner with researchers conducting vascular screenings as part of their clinical trials to afford those in the community services not yet rendered in hospital settings.

Policy implications include integrating this screening tool into clinical care for at risk individuals. The United States Task Force Services (USTFS) gives recommendations for early vascular screenings, especially if risk factors are present (Sugerman, 2013). Moving forward, we not only include where we live, but also how we live as a social determinant of health.

## Figures and Tables

**Table 1.** Demoeranhics of Study Population

Variable	N=67 N (%)
**C1 Arterial Screening**	
Normal/Borderline	18(39.13)
Abnormal	28(60.87)
**C2 Arterial Screening**	
Normal/Borderline	18(39.13)
Abnormal	28(60.87)
**Risk Factors**	
No Risk Factors	34(50.75)
Yes Risk Factors	33(49.25)
**Family CVD**	
No Heart Condition	7(10.45)
Yes Heart Condition	60(89.55)
**Mom-Dad CVD**	
No Heart Condition	8(11.94)
Yes Heart Condition	59(88.06)
**Grandparent CVD**	
No Heart Condition	43(64.18)
Yes Heart Condition	24(35.82)
**Sibling CVD**	
No Heart Condition	66(98.51)
Yes Heart Condition	1(1.49)
**Level of Education**	
High School/GED	6(8.95)
College	23(35.33)
Graduate/PhD	38(56.72)
**Living Arrangement**	
Living Alone	20(29.85)
Living with Spouse/Partner or Relative	47(70.15)

**Table 2.** Family History and Factors by Arterial Screening C1 and C2

Arterial Elasticity Screening C1			p-value
**Family CVD**	Normal/Borderline	Abnormal	
No Heart Condition	4(67)	2(33)	0.15
Yes Heart Condition	14(35)	26(65)	
**Mom-Dad CVD**			
No Heart Condition	4(57)	3(43)	0.25
Yes Heart Condition	14(36)	25(64)	
**Living Arrangement**			
Living Alone	5(29)	12(71)	0.36
Living with Spouse/Partner or With Relative	13(45)	16(55)	
**Tobacco Use**			
Current or Past	11(35)	22(65)	0.21
Never	7(54)	6(46)	
Arterial Elasticity Screening C2			
**Family CVD**			
No Heart Condition	1(17)	5(83)	0.22
Yes Heart Condition	17(42)	23(58)	
**Mom-Dad CVD**			
No Heart Condition	1(14)	6(85)	0.12
Yes Heart Condition	17(44)	22(56)	
**Living Arrangement**			
Living Alone	3(18)	14(82)	0.03
Living with Spouse/Partner or With Relative	15(52)	14(48)	

**Table 3.** Multivariable Analysis of Association of Risk Factors and Abnormal C1 and C2 Measures among African American Women in the Southeast Region of the United States, 2015

	C1 Arterial Elasticity			C2 Arterial Elasticity		
	Odds Ratio	95% Confidence Interval	p-value	Odds Ratio	95% Confidence Interval	p-value
**Risk Factors**						
Risk Factors Absent	1.00			1.00		
Risk Factors Present	0.902	0.240-3.386	0.878	0.894	0.245-3.262	0.8651
**Family CVD**						
No Heart Condition	1.00			1.00		
Yes Heart Condition	7.894	0.928-67.142	0.058	0.350	0.031-3.933	0.3948
**Living Arrangement**						
Living Alone	1.00			1.00		
Living with Spouse/Partner or with Relative	0.350	0.079-1.543	0.166	0.222	0.050-0.985	0.0411
**High Lipid Cholesterol**						
No Heart Condition	1.00			1.00		
Yes Heart Condition	0.526	0.135-2.045	0.354	1.40	0.368-5.323	0.7912
